# Effects of rapid recruitment and dissemination on Covid-19 mortality: the RECOVERY trial

**DOI:** 10.12688/f1000research.25842.2

**Published:** 2020-12-23

**Authors:** Catherine Knowlson, David J. Torgerson

**Affiliations:** 1 York Trials Unit, Department of Health Sciences, University of York, York, YO10 5DD, UK

**Keywords:** Covid-19, RECOVERY trial, recruitment

## Abstract

The RECOVERY trial is a large multi-armed, adaptive randomised controlled trial of treatments for Covid-19.  It has rapidly recruited and demonstrated that hydroxychloroquine is ineffective in reducing mortality for hospitalised patients, whilst dexamethasone significantly reduces mortality among those patients using supplemental oxygen or on a ventilator.  We estimate that the speed of recruitment and dissemination has probably decreased mortality in the UK by at least 200 hospitalised patients in the first month since the British Prime Minister announced the results.  Despite its impressive speed, the trial only recruited about 10-15% of eligible patients, with recruitment rates ranging between 3% to 80% at participating hospitals.  Had the trial recruited 50% of the eligible patients then our analysis suggests that more than 2,000 additional lives could have been saved.  In a pandemic, rapid recruitment with high centre recruitment is absolutely essential to reduce deaths.  Methods of improving site specific recruitment rates need investigating urgently.

## Introduction

The RECOVERY randomised controlled trial (RCT) is a world-leading study of potential treatments for Covid-19 patients. It is large, simple, adaptive and multi-armed, allowing the investigators to test several treatments at the same time and quickly close trial arms if one of the treatments were found to be effective or ineffective. Importantly, this means the results can be rapidly disseminated to patients, clinicians and policy makers. The trial initially randomised Covid-19 patients admitted to hospital to one of five treatments: lopinavir-ritonavir (an HIV treatment); hydroxychloroquine; dexamethasone; azithromycin or usual care. The protocol was kept simple and flexible to allow “ *a broad range of patients to be enrolled in large numbers*” (RECOVERY study protocol ^1^). Uniquely, the trial started recruiting nine days after submission of its protocol ^2^.

The trial has rapidly produced some key findings on the effectiveness and ineffectiveness of potential Covid-19 treatments. Its earliest, and somewhat overlooked, finding that hydroxychloroquine was ineffective (and probably harmful) to Covid-19 patients was important given that it has been widely promoted and used ^3^. If the point estimate, of harm, in the hydroxychloroquine comparison is correct, then many lives will be saved worldwide by its, hopeful, reduction in use. Most recently the trial has demonstrated that lopinavir-ritonavir is also ineffective ^4^. The most widely publicised finding from the RECOVERY trial, however, was that of the dexamethasone arm, which statistically significantly reduced mortality among Covid-19 patients at 28 days after randomisation ^3^. This important result was demonstrated in less than three months after the trial was set-up. Within 81 days the trialists recruited 175 hospitals and enrolled 11,303 participants with 9,355 eligible for the dexamethasone comparison ^3^. This remarkable trial will lead to many hundreds of lives saved across the UK and the world and it is a tribute to the investigators and all those who took part either as participants, clinicians or researchers.

The RECOVERY trial is unique in its rapid recruitment and the speed at which it reported its first findings. Most trials, however, recruit relatively poorly and slowly, and therefore do not report their results in a timely fashion. RECOVERY did not recruit slowly but arguably it did recruit poorly. It has been reported that the overall recruitment rate to RECOVERY was 15% ^2^ of Covid-19 inpatients, with participating hospital recruitment ranging between 3% and 80% of eligible patients being recruited ^2^. In this respect, RECOVERY exhibited similar characteristics of the ‘typical’ non-Covid trial undertaken within an NHS setting: some recruitment sites enrol a very high proportion of eligible patients while others recruit relatively low numbers. Indeed, it is rare for all sites, or the majority, to consistently recruit a high proportion of eligible participants ^5, 6^.

For the ‘standard’ trial (and for RECOVERY) to ensure rapid recruitment in the presence of poor average site recruitment, many more sites have to be enrolled in the study than would be required if there was high recruitment in all clinical sites, or recruitment of the target number takes longer than expected. However, if all sites could recruit the same proportion of eligible participants as the best recruiting site then trials would be finished more rapidly. This would have the benefits of reducing the overall cost of the trial and, most importantly, would improve patients’ health and save more lives. In ‘normal’ times this trade-off in lost lives and reduced quality of life, due to low recruitment, is not identified because either the data are not routinely collected (e.g., quality of life) or it is not collated so that it can be quantified. Slow or poor recruitment is even more catastrophic during a pandemic as there is a brief window of opportunity to recruit and complete a trial to enable infected patients to benefit from novel treatments. Therefore, whilst the clinical trials community in the UK has led the world in rapid, large and effective clinical trials to identify new treatments for Covid-19 there is still room for improvement. In this paper we look at the potential impact of the RECOVERY results on the numbers of patients surviving since the dexamethasone results were reported and then discuss the likely consequences of the RECOVERY trial’s ability to recruit only 15% ^2^ or less of the UK’s hospitalised Covid-19 patients into the trial.

## Methods

### First month mortality impact of RECOVERY results

To examine the actual impact of the RECOVERY trial on lives saved and its ‘potential’ impact had recruitment been even more swift, we used UK estimates of hospital admissions due to Covid-19 ^7– 10^. We assumed that the proportion of patients that were eligible was the same as described in the RECOVERY trial, as well as the proportions on oxygen and ventilation. We used admissions data from the 16 ^th^ June 2020 (date of the release of the trial results) until 15 ^th^ July 2020. However, in line with the RECOVERY results we assumed that 24% of admitted patients did not need either oxygen or ventilator support so would not be offered the dexamethasone.

### More rapid recruitment to the RECOVERY trial

The RECOVERY trial recruited 15% of patients with Covid-19 in UK hospitals. There was a huge variation in recruitment rates across the trial, which ranged from 3% to 80% of eligible participants. Recruitment started on the 19 ^th^ March 2020 with rapid accrual of hospitals (132 participating hospitals by 3 ^rd^ April) and by the 8 ^th^ of June 2020 (with 175 hospitals open to recruitment), 11,303 patients had been randomised in total. Of these, 9,355 were randomised into the steroid comparison so this part of the study closed to recruitment ^3^. Assuming an overall 15% recruitment rate, then this implies there were 75,353 patients with Covid-19 in UK hospitals during the recruitment period (although routine statistics suggest that there had been 114,935 Covid-19 admissions across the UK by this date ^7– 10^). Making the following assumptions we can estimate the possible loss of life by not recruiting a greater proportion of Covid-19 patients. In our following calculations we assume that on average 50% of eligible patients would take part in the RECOVERY trial if asked. Therefore, to enrol 11,303 patients then we would have to identify 22,606 patients admitted to NHS hospitals with COVID-19. We estimate this target would have been reached by the 1 ^st^ April (as 24,978 COVID-19 patients had been admitted by this point ^7– 10^). The RECOVERY trial’s preliminary results were released by the British Prime Minister eight days after recruitment was completed, which would have taken us to the 9 ^th^ April 2020 (by which time 48,075 patients had been admitted to hospital in the UK). Between the 9 ^th^ April 2020, when the results could have been available, and the 15 ^th^ July 2020 there were 77,310 patients admitted with Covid-19 ^7– 10^. To estimated the number of lives which could have been saved by the earlier completion of the dexamethasone arm, we made the following assumptions base on the RECOVERY trial results: that 83% of admitted patients had no contraindications to dexamethasone, and that 24% of admitted patients did not need either oxygen or ventilator support so would not be offered the dexamethasone.

## Results

In Table 1 we show the estimated lives saved in this first month of dexamethasone being made available to all eligible patients (assuming that all hospitals implemented the guidelines without delay). In this month there were approximately 6,980 patients admitted to hospital with Covid-19, which equates to an estimated 5,793 patients who had no contraindications for dexamethasone treatment. Table 1 shows that in just over a month more than 200 extra patients in the UK survived in hospital due to wider use of dexamethasone.

**Table 1.** Estimated numbers of additional patients who survived up to 15 ^th^ July due to RECOVERY.

Status	Proportion in each status as per RECOVERY Trial (Numbers admitted from 16 ^th^ June to 15 ^th^ July (6,980) of which 83% are eligible for dexamethasone) N = 5,793	Estimated deaths despite dexamethasone	Estimated deaths without dexamethasone
No Oxygen	24% (1,390)	14.0% * (195)	14.0% (195)
Oxygen alone	60% (3476)	22.0% ** (765)	26.2%(911)
Ventilation	16% (927)	29.1% ** (270)	41.4% (384)
Total deaths		1,230	1,490
Additional lives saved		260	

*Assumes steroids are not given to hospitalised but not oxygenated patients as per the results from the RECOVERY trial. **Adjusted rather than observed differences between groups are used, which are 12.3 and 4.2% reduction in 28-day mortality for ventilated and oxygen supported patients, respectively.

In Table 2, using the RECOVERY data we have estimated the potential benefit had all the participating hospitals recruited 50% of their eligible patients to RECOVERY (which should be achievable as clinical experience suggests that the vast majority of patients were happy to be included in the trial ^2^, although we are assuming there are no other large Covid-19 studies which would have caused competition for participants) and the dexamethasone recruitment was halted at 9,355 patients and the results were available by the 9 ^th^ April.

**Table 2.** Estimated additional patients who survived up to 15 ^th^ July with 50% recruitment to RECOVERY.

Status	Proportion in each status as per the RECOVERY Trial (Numbers admitted from 9 ^th^ April to 15 ^th^ July (77,310) of which 83% are eligible for dexamethasone) N = 64,167	Steroid deaths	Usual care deaths
No Oxygen	24% (15,400)	14.0% * (2,156)	14.0% (2,156)
Oxygen alone	60% (38,500)	22.0% ** (8,470)	26.2%(10,087)
Ventilation	16% (10267)	29.1% ** (2988)	41.4% (4,251)
Total deaths		13,614	16,494
Additional lives saved		2,880	

*Assumes steroids are not given to hospitalised but not oxygenated patients as per the results from the RECOVERY trial. **Adjusted rather than observed differences between groups are used, which are 12.3 and 4.2% point reduction in 28-day mortality for ventilated and oxygen supported patients, respectively.

The table shows that by not achieving the best recruitment which some UK hospitals are capable of means around 2,880 patients died unnecessarily.

## Discussion

There is a need to complete and report all trials more quickly. This is especially the case in a pandemic. A reason why the RECOVERY trial could be done in the UK is due to the strong research infrastructure and having a national health service. However, we could do better. During the height of the pandemic, government advisors in the daily briefing encouraged patients and their doctors to take part in clinical trials. Whilst some hospitals recruited a remarkable 80% of eligible patients many did less well or did not take part ^2^. If some hospitals can recruit such high proportions of participants, then the majority should be able to do so. The differences in recruitment between sites is dramatic, and it would be interesting to find out why. If further information was released from the RECOVERY team, it could be determined whether there was a correlation between the number of COVID-19 admissions (and therefore reduced capacity of site teams) and poor recruitment. We understand that hospitals will be under more pressure than normal, especially when the number of cases are high, which may reduce their ability to recruit. We suspect that the reason for the disparity is unlikely to be this simple, as our experience, across a range of clinical areas, is that even before the pandemic trials have often had a disproportionate recruitment across sites. It would be beneficial to trial management teams to find out why some sites are more efficient at recruiting (such as a greater trust-wide emphasis on research) and whether this information can be used to transform low recruiting sites to reach their full potential. This remains a key area of research which should be explored in the future.

In addition to where the patients are being recruited to the RECOVERY trial, it is important to consider who are the patients taking part and whether there was any bias about who was approached. There was a difference between sexes taking part in the Dexamethasone comparison as 64% were men and 36% were women, however this fits with the finding that men are more likely to be seriously affected by COVID-19 and therefore more men are hospitalised with the condition ^11^. While the ethnicity of participants in the dexamethasone arm of the study was not published, those involved in the another comparison of the RECOVERY trial, between laponavir-ritonavir and usual care, were 15–18% Black, Asian and minority ethnic, compared with 74–77% White (the remaining 8% being unknown) ^12^, which fits with the ethnicity proportions in the UK ^13^. The baseline characteristics therefore do not suggest a major disparity between the patients recruited and their distribution in the general population.

If there are no proven treatments available yet, we would argue that the best care for affected patients would be to offer participation in a study to help identify an effective treatment. If there is a second wave of the disease over the winter then measures need to be put into place to ensure that all eligible patients are offered the chance to take part in a clinical trial: swift action in recruitment will save more lives.

There has been some criticism of the RECOVERY trialists for reporting their results by press conference rather than in a peer reviewed journal ^2^. The peer-reviewed paper published in the *New England Journal of Medicine* ^3^ on July 17 ^th^ 2020 had only trivial differences from the basic data released on the 16 ^th^ June 2020. Had the trialists waited for the peer reviewed paper to be published before having a press conference then it is likely over 200 patients in the UK would have died, plus many more internationally. Consequently, the rapid dissemination of results, in our view, was justified.

Whilst this paper has focused just on the dexamethasone results we must not forget the potentially harmful effects noted in the use of hydroxychloroquine ^14^. Results from the RECOVERY trial showed the use of hydroxychloroquine led to a combined increase in mortality and ventilator use among hospitalised patients. Whilst ‘off label’ use of this drug for covid-19 is likely be small in the UK, the Food and Drug Administration (FDA) in the USA gave an emergency use authorisation for Covid-19 patients on the basis if little robust evidence ^15^. This would have led to widespread use in the USA. The RECOVERY trial’s results are likely to have reduced the use of this drug and led to a reduction in mortality and morbidity, which is beyond the scope of this paper to estimate.

## Conclusions

Rapid recruitment and dissemination in the RECOVERY trial has, we estimate, saved at least 200 lives in the UK in first month since the trial’s results were released. However, we have estimated that the number lives saved, had the recruitment rate been at least 50% of eligible patients, would have been an order of magnitude greater.

## Data availability

### Underlying data

All data underlying the results are available as part of the article and no additional source data are required.
