# Supplementary material for: Effects of rapid recruitment and dissemination on Covid-19 mortality: the RECOVERY trial
Source: F1000Res. 2020 Dec 23;9:1017. Originally published 2020 Aug 21. [Version 2] doi: 10.12688/f1000research.25842.2 (PMC7783530; doi:10.12688/f1000research.25842.2)
Supplement: Peer review history

# Peer review: Reviewer response for version 2

**DOI:** 10.5256/f1000research.31480.r76357

**Published:** 2021-01-04

**Authors:** Carlos Chaccour

**Affiliations:** 1 Instituto de Salud Global de Barcelona, Barcelona, Spain; 2 Universidad de Navarra, Pamplona, Spain; 3 Ifakara Health Institute, United Republic of Tanzania, Tanzania

The authors have addressed my minor comments.

Is the topic of the opinion article discussed accurately in the context of the current literature?

Yes

Are arguments sufficiently supported by evidence from the published literature?

Yes

Are all factual statements correct and adequately supported by citations?

Yes

Are the conclusions drawn balanced and justified on the basis of the presented arguments?

Partly

Reviewer Expertise:

Internal medicine, Infectious diseases, epidemiology

I confirm that I have read this submission and believe that I have an appropriate level of expertise to confirm that it is of an acceptable scientific standard.

# Peer review: Reviewer response for version 2

**DOI:** 10.5256/f1000research.31480.r76358

**Published:** 2021-01-04

**Authors:** Heidi Gardner

**Affiliations:** 1 Health Services Research Unit, University of Aberdeen, Aberdeen, UK

Thank you for making revisions to this article based on my feedback, it's a great piece of work and one that I'm looking forward to seeing built upon in the future.

Is the topic of the opinion article discussed accurately in the context of the current literature?

Yes

Are arguments sufficiently supported by evidence from the published literature?

Yes

Are all factual statements correct and adequately supported by citations?

Yes

Are the conclusions drawn balanced and justified on the basis of the presented arguments?

Yes

Reviewer Expertise:

Clinical trials methodology, participant recruitment, mixed methods research, inclusivity in trials, trial efficiency

I confirm that I have read this submission and believe that I have an appropriate level of expertise to confirm that it is of an acceptable scientific standard.

# Peer review: Reviewer response for version 1

**DOI:** 10.5256/f1000research.28519.r74832

**Published:** 2020-12-09

**Authors:** Heidi Gardner

**Affiliations:** 1 Health Services Research Unit, University of Aberdeen, Aberdeen, UK

Thank you for requesting that I review this interesting and very timely article. I enjoyed reading this, as usual from these authors the manuscript is well written, robustly reported, and refreshingly concise.

 The range of recruitment rates from 3% to 80% was shocking to me, and I'd like to see more detail in this if possible. Are the authors able to break these rates down to individual hospitals or health boards? I'd be interested to know if the low recruiters had high rates of COVID and vice versa to see if there's a correlation. More broadly this information could provide insights into how various sites view trials - are they a priority? If not, why not? If so, what's contributing to that culture and how can we replicate it in other sites?

 The other thing that I feel is lacking in this paper is discussion around WHO the participants are. Recruiting is all well and good, but if RECOVERY recruited only white British people and no minorities, then that's clearly a problem - particularly given the disparities around the impact of COVID on different ethnic minority groups in our society. The fact that RECOVERY doesn't appear to have collected data on the ethnicity of participants is problematic, and deserves a comment here.

 The implications of this work are potentially wide-ranging, and have got me thinking about what research area(s) should be focused on to ensure that the day-to-day usual trials that are run in the UK are as effective (and quick) as they can be. I'd like to see what the authors think about this. Should we be thinking about engagement with the public, involvement of public and patient partners, research into how the option to take part in a trial could potentially be a part of routine care? The fact that patients have died as a result of poor recruitment in RECOVERY is a fantastic starting point for discussion - the numbers are stark, but what to do with them now?

 Overall, this is an important and thought-provoking article, and one that should act as a wake-up call to those of us working to improve trials. There is much work to be done in this area.

Is the topic of the opinion article discussed accurately in the context of the current literature?

Yes

Are arguments sufficiently supported by evidence from the published literature?

Yes

Are all factual statements correct and adequately supported by citations?

Yes

Are the conclusions drawn balanced and justified on the basis of the presented arguments?

Yes

Reviewer Expertise:

Clinical trials methodology, participant recruitment, mixed methods research, inclusivity in trials, trial efficiency

I confirm that I have read this submission and believe that I have an appropriate level of expertise to confirm that it is of an acceptable scientific standard, however I have significant reservations, as outlined above.

# Response

**Published:** 2020-12-15

**Authors:** David Torgerson

**Affiliations:** University of York, UK

With respect to the second reviewer (Heidi Gardener) we very much agree with you that it would be interesting to find out what the differences were between the participating hospitals that caused the wide range in recruitment rate, as this would be incredibly valuable for current and future trials. Unfortunately, as we are not involved in the RECOVERY trial we do not have access to data broken down by site to determine whether there is a correlation between poor recruitment and the number of covid admissions. You have definitely touched upon an interesting area for consideration, around how different sites view trials. Although this was outside the scope of this study, it is certainly something we would be interested in researching in the future.

 You brought up another excellent point about who the participants are that participated in the trial.  In two of the three published RECOVERY papers these data are not reported.  However, one of the papers does report the proportion of participants from ethnic minorities and we have updated the paper to include a section on this in the discussion, which as far as we can see there does not seem to be a disparity.  However, unfortunately we are unable to look at BAME admission rates across the participating sites to know for sure if there is any disparity as we do not have access to these data. Again, this is a potential area of future research if the RECOVERY team can provide this information.

 It would be fantastic if we could improve recruitment rates at those sites which do not deliver to target. We think that there is unlikely to be a single factor that is involved, and it will very much depend on individual sites and reasons may change over time too (e.g. site capacity, competing studies, Principal Investigator engagement). Indeed, we hope that the increased media attention on clinical trials recently will encourage  more patients to seek participation in trials, however we think that the biggest effect would be at a site level as we believe that all patients should be given the opportunity to take part in a trial.

 We have addressed these issues in the updated version of our paper.

# Peer review: Reviewer response for version 1

**DOI:** 10.5256/f1000research.28519.r74833

**Published:** 2020-11-27

**Authors:** Carlos Chaccour

**Affiliations:** 1 Instituto de Salud Global de Barcelona, Barcelona, Spain; 2 Universidad de Navarra, Pamplona, Spain; 3 Ifakara Health Institute, United Republic of Tanzania, Tanzania

In this opinion piece, Knowlson and Torgerson analyze the recruitment rate and recruitment success of the RECOVERY trial. They point to a large variation between sites ranging from 15 to 80% of all eligible patients and argue that a much faster completion of the trial was feasible if all sites had recruited at least 50% of eligible patients. The authors then proceed to estimate the lives lost due to poor recruitment delaying trial completion. This analysis is based on the proven efficacy of dexamethasone. 

 The opinion piece is indeed provoking and well thought. The introduction is well written and sets the stage appropriately. The methods and assumptions are described at large and the results mostly support the conclusion.

 As an opinion piece, I think it could further improve by mentioning other potential causes for slow/poor recruitment, such as whether hydroxychloroquine, azithromycin or any of the RECOVERY interventions were used outside the trial. 

 The authors make clear that their conclusion is based on the effect of dexamethasone and I think this sufficiently illustrates the point. However, as much as RECOVERY served to support the scale-up of dexamethasone, it served to reduce the compassionate use of hydroxychloroquine which often comes with additional risk of harms. Additional consideration could be given to the lives saved by a reduction in the use of hydroxychloroquine. 

Minor:

- There is a zero missing in the phrase: “from the 16 ^th^ June 2020 (date of the release of the trial results) until 15 ^th^ July 202:”.
- The assumption: "83% of patients without contraindications and 24% not needing oxygen or ICU" is mentioned twice.

Is the topic of the opinion article discussed accurately in the context of the current literature?

Yes

Are arguments sufficiently supported by evidence from the published literature?

Yes

Are all factual statements correct and adequately supported by citations?

Yes

Are the conclusions drawn balanced and justified on the basis of the presented arguments?

Partly

Reviewer Expertise:

Internal medicine, Infectious diseases, epidemiology

I confirm that I have read this submission and believe that I have an appropriate level of expertise to confirm that it is of an acceptable scientific standard.

# Response

**Published:** 2020-12-15

**Authors:** David Torgerson

**Affiliations:** University of York, UK

With respect to the first reviewer (Carlos Chaccour) we have undertaken the minor corrections he suggested in his review.  We have also added an additional paragraph in the discussion regarding the point he made about the impact of the RECOVERY findings on off label hydroxychloroquine use, which, worldwide, would also have an impact on reducing harm.  We include this in our updated version of the paper.
